# Therapeutic potential of targeting cell division cycle associated 5 for oral squamous cell carcinoma

**DOI:** 10.18632/oncotarget.6148

**Published:** 2015-10-19

**Authors:** Norihiko Tokuzen, Koh-ichi Nakashiro, Hiroshi Tanaka, Kazuki Iwamoto, Hiroyuki Hamakawa

**Affiliations:** ^1^ Department of Oral and Maxillofacial Surgery, Ehime University Graduate School of Medicine, Shitsukawa, Toon, Ehime, Japan

**Keywords:** cell division cycle associated 5 (CDCA5), oral squamous cell carcinoma (OSCC), cell cycle, prognosis, molecular targeted therapy

## Abstract

Molecularly targeted drugs are used in the treatment of a variety of malignant tumors, but this approach to developing novel therapies for oral squamous cell carcinoma (OSCC) has lagged behind the progress seen for other cancers. We have attempted to find appropriate molecular targets for OSCC and identified cell division cycle associated 5 (CDCA5) as a cancer-related gene which was overexpressed in all the human OSCC cells tested by microarray analysis. In this study, we investigated the expression and function of CDCA5 in OSCC. First, we confirmed that CDCA5 was overexpressed in 4 human OSCC cell lines by quantitative RT-PCR and Western blotting. We then tested the effect of synthetic small interfering RNAs specific for CDCA5 on the growth and invasion of human OSCC cells. Knockdown of CDCA5 markedly inhibited the growth of OSCC cells *in vitro* and *in vivo*. We also examined the expression of CDCA5 protein in 80 cases of OSCC immunohistochemically and found a significant association between CDCA5 expression levels and overall survival. These results suggest that CDCA5 functions as a critical gene supporting OSCC progression and that targeting CDCA5 may be a useful therapeutic strategy for OSCC.

## INTRODUCTION

Oral squamous cell carcinoma (OSCC) is estimated 263,000 new cases and 127,000 deaths in 2008 worldwide [1]. OSCC has a high potential to invade local tissue and metastasize to lymph nodes, and has a mortality rate of approximately 50% within 5 years [2]. Despite our increasing knowledge of OSCC pathogenesis and advances in chemotherapy, radiotherapy, and surgery, little improvement in the relative survival rate of patients with OSCC has been observed in the past several decades [2]. Therefore, novel strategies based on a greater understanding of the pathogenesis of OSCC are needed for the development of improved therapeutic approaches.

Cancer cells acquire abnormalities in multiple oncogenes and tumor-suppressor genes. Overexpression and constitutive activation of some of these oncogenes support the proliferation, invasion, and metastasis of cancer cells. However, this dependence on oncogenes for maintaining the cancer phenotype also provides molecular targets, which can be exploited in cancer therapy [3]. Recent studies of human malignancies have shown that it is possible to use pharmacological agents that inactivate oncogenes to treat some types of human cancer. For example, imatinib, which targets breakpoint cluster region-abelson (BCR-ABL), is used to treat patients with chronic myelogenous leukemia [4] and crizotinib, which targets anaplastic lymphoma kinase (ALK), is used to treat patients with ALK-positive non-small cell lung cancer [5]. Although targeting oncogenes in this way has provided novel therapeutic opportunities, the development of molecular targeted therapy for OSCC has lagged behind other cancers. Therefore, we have attempted to identify appropriate molecular targets for OSCC. In a previous study, we used microarray analysis and Ingenuity Pathway Analysis (IPA) to identify 465 cancer-related genes that were overexpressed in all the human OSCC cell lines examined [6]. Among these genes, we identified cell division cycle associated 5 (CDCA5) as a substrate of the anaphase-promoting complex (APC) and as a regulator of sister chromatid cohesion in HeLa cells [7-9]. CDCA5 protein is degraded through Cdc20 homolog 1 (Cdh1)-activated APC (APC^cdh1^)-dependent ubiquitination in the G1 phase and is required for sister chromatid cohesion in the S and G2 phases [7]. Furthermore, CDCA5 has been reported to be overexpressed in the majority of human lung cancers and urothelial cancers and to play a critical role in carcinogenesis [10-13].

In this study, we investigated the expression and function of CDCA5 in OSCC to clarify whether targeting CDCA5 is likely to be a promising strategy for the treatment of OSCC.

## RESULTS

### Overexpression of CDCA5 in human OSCC cells

**Figure 1:**
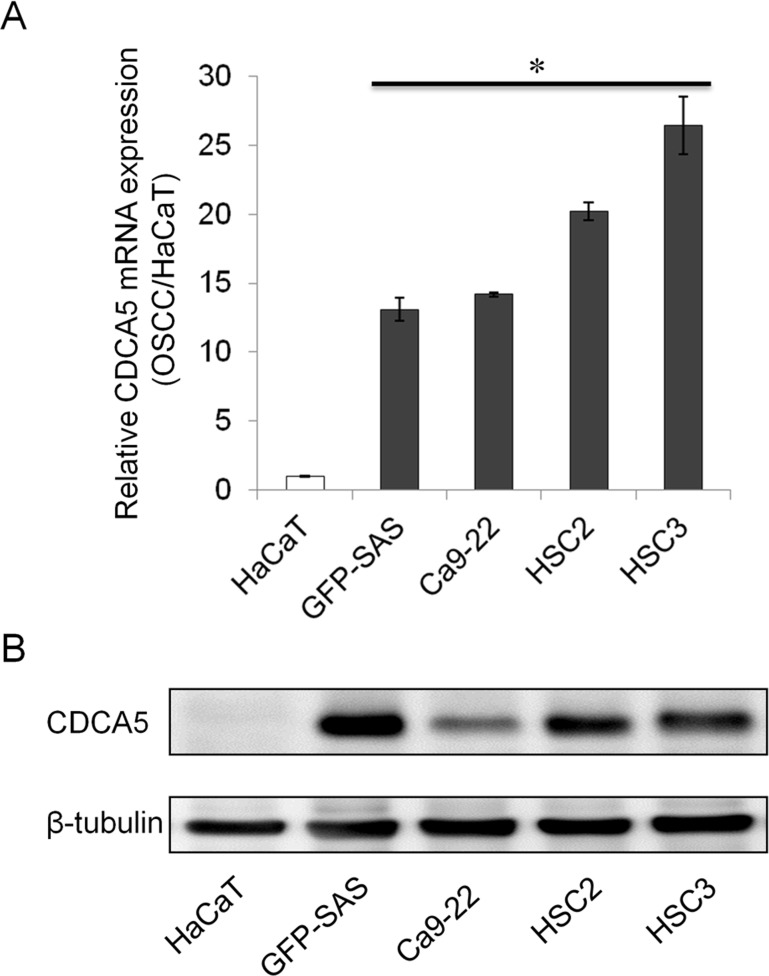
Overexpression of CDCA5 in human OSCC cells **A.** Expression of CDCA5 mRNA in 4 human OSCC cell lines and a human immortalized non-neoplastic keratinocyte cell line (HaCaT) was evaluated by qRT-PCR. Expression levels are shown relative to HaCaT cells. *, *p* < 0.01 compared to HaCaT. **B.** Western blots showing the expression of CDCA5 protein compared to β−tubulin in OSCC cells.

We confirmed the expression of CDCA5 mRNA in 4 human OSCC cell lines (green fluorescent protein (GFP)-SAS, Ca9-22, HSC2, and HSC3) by real-time quantitative RT-PCR (qRT-PCR). High levels of CDCA5 mRNA expression were observed in all OSCC cell lines compared with the human immortalized non-neoplastic keratinocyte cell line, HaCaT (Figure 1A). Subsequently, we examined the expression levels of CDCA5 protein by Western blot analysis and detected its abundant expression in OSCC cells, whereas its expression was hardly detectable in HaCaT cells (Figure 1B). These results showed that both CDCA5 mRNA and protein were overexpressed in human OSCC cells.

### Effect of CDCA5 suppression on the growth of human OSCC cells *in vitro*

**Figure 2:**
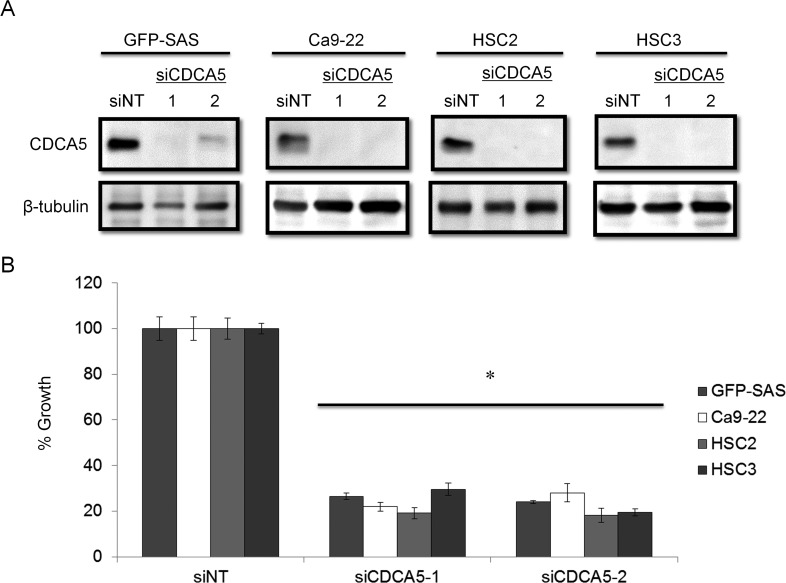
Knockdown of CDCA5 in human OSCC cells by siRNA **A.** Western blots showing the effect of RNAi on CDCA5 expression. Two different siCDCA5s at 10 nM were transfected into human OSCC cell lines GFP-SAS, Ca9-22, HSC2, and HSC3 with Lipofectamine RNAiMAX. **B.** The effect of transfecting siCDCA5 into human OSCC cell lines on their growth, evaluated using WST-8 assays. *, *p* < 0.01 Growth is expressed relative to the same cell cultures transfected with an untargeted, siNT.

We clarified the function of CDCA5 in the proliferation and invasiveness of human OSCC cells, by transfecting them with synthetic small interfering RNA (siRNA) specific for CDCA5 (siCDCA5) at a concentration of 10 nM. Synthetic siCDCA5 suppressed the expression of CDCA5 protein (Figure 2A). When we then examined the effect of siCDCA5 on the growth and invasion of human OSCC cells, we found that CDCA5 knockdown significantly inhibited cell growth by 50-81% (Figure 2B) but not invasiveness (data not shown), compared to an untargeted siRNA (siNT).

### Role of CDCA5 in the cell cycle in human OSCC cells

**Figure 3:**
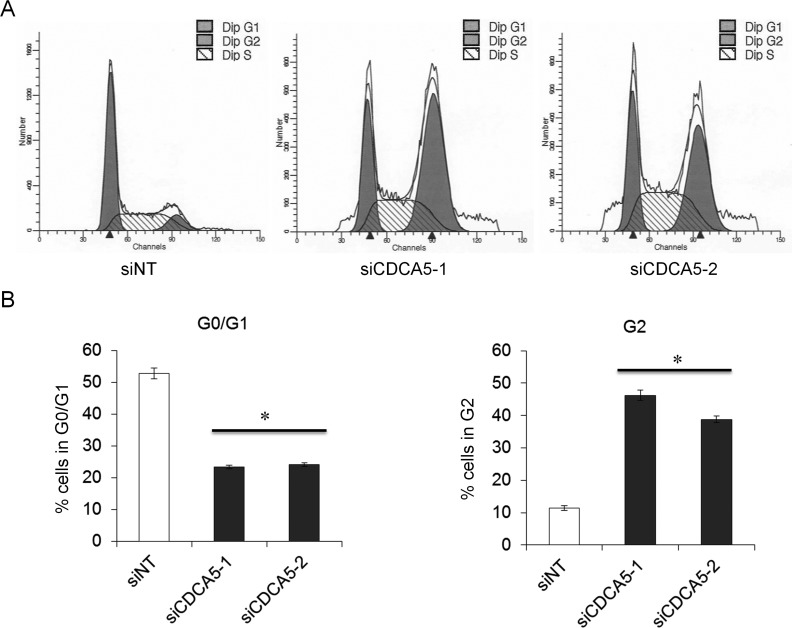
Role of CDCA5 in the cell cycle of human OSCC cells **A.** Flow cytometry profiles showing numbers of GFP-SAS cells in G1, G2, and S phase 48 h after transfection with different siRNAs at 10 nM. **B.** Histograms showing the percentage of GFP-SAS cells in different stages of the cell cycle 48 h after transfection with siRNAs. *, *p* < 0.01 compared to control culture.

CDCA5 has been reported to have an important role in the cell cycle [7-9]. We therefore analyzed the effect of siCDCA5 on the cell cycle by flow cytometry using GFP-SAS cells. After the cells had been treated with 10 nM siCDCA5 for 48 h, significantly fewer cells were in G0/G1 phase and significantly more were in G2 phase, compared to cells treated with siNT (Figure 3A, 3B). The data indicated that the anti-proliferative effect of siCDCA5 was due to an arrest in G2. Ca9-22, HSC2, and HSC3 cells also showed the similar results (data not shown).

### Effect of siCDCA5 on the *in vivo* growth of human OSCC cells

**Figure 4:**
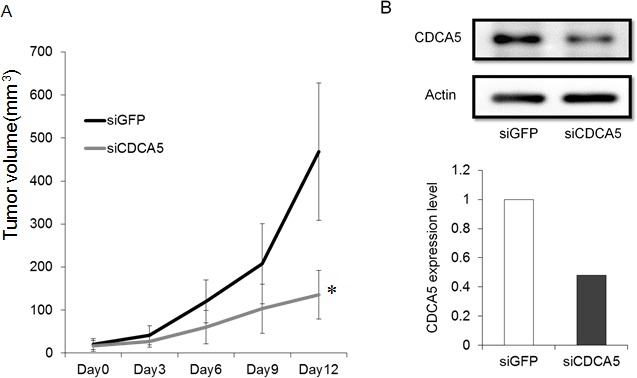
Effect of siCDCA5 on the *in vivo* growth of human OSCC cells **A.** GFP-SAS cells at 2 × 10^6^/50 μl were injected subcutaneously into the flanks of nude mice with an equal volume of Matrigel®. Synthetic siCDCA5/atelocollagen complexes were injected around the tumor every 3 days. Tumor growth was measured and volumes calculated until the tumors were excised on day 13. *, *p* < 0.01 compared to control culture. **B.** CDCA5 protein expression was assessed in excised tumors by Western blotting (upper panel) and densitometry (lower panel).

We assessed the growth inhibitory effect of siCDCA5 *in vivo* using a mouse model. We used GFP-SAS cells for this *in vivo* assay because only these cells, of the four lines we used, showed stable tumorigenicity. We administered siCDCA5/atelocollagen complexes into the subcutaneous spaces around the tumors every 3 days. We found that these complexes significantly reduced the size of subcutaneously xenografted GFP-SAS tumors, compared with the control group treated with synthetic siRNA specific for GFP (siGFP)/atelocollagen complexes (Figure 4A). Furthermore, the expression of CDCA5 in excised tumor tissue was markedly suppressed, by 53%, in the group treated with siCDCA5 (Figure 4B). During the administration of siCDCA5, no reduction in either food intake or body weight was seen in the mice. We also examined the expression of interferon response genes such as interferon stimulated gene factor 3 γ (ISGF-3γ), 2′, 5′-oligoadenylate synthetase 2 (OAS2), and interferon-induced myxovirus resistance protein 1 (MX1) in liver and lung tissues from mice by qRT-PCR. There was no significantly induction of these genes by treatment with siGFP or siCDCA5/atelocollagen complex (data not shown).

### Effect of targeting CDCA5 in primary human OSCC cultured cells

**Figure 5:**
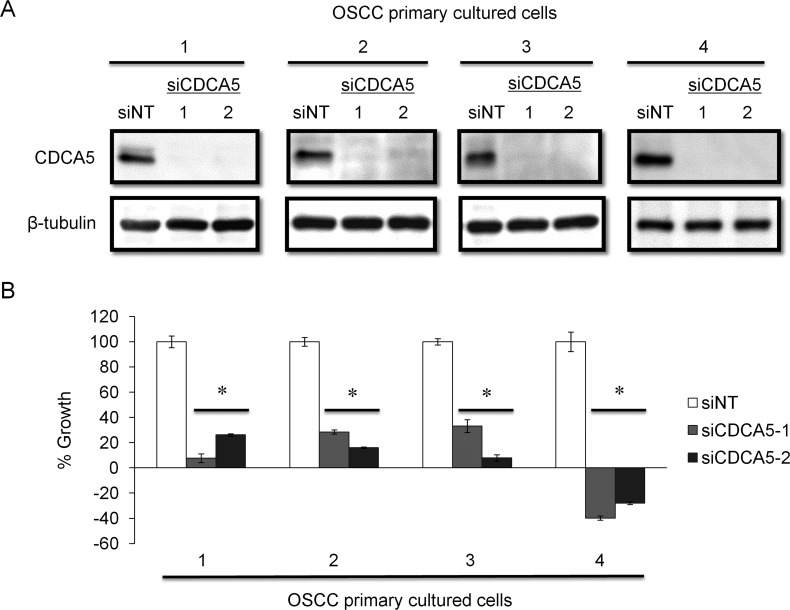
Effect of targeting CDCA5 in human OSCC primary cell cultures **A.** OSCC primary cultured cells were derived from lower gingiva of 55 year-old male (1), lymph node metastasis of 55 year-old male (2), lower gingiva of 80 year-old female (3), and skin metastasis of 61 year-old male (4), respectively. These cells were transfected with 10 nM siCDCA5 in Lipofectamin RNAiMAX. The effects of RNAi were analyzed by Western blotting. **B.** Cell growth was evaluated 72 h after transfection using WST-8 assays. *, *p* < 0.01 compared to control cultures.

To confirm the usefulness of targeting CDCA5 in OSCC, we established primary cell cultures from newly resected tumor tissues from patients with OSCC. These primary cultured cells were derived from two lower gingival tumors, a lymph node metastasis, and a skin metastasis. When we tested the effect of siCDCA5 on these primary cultures, as with the established human OSCC cell lines, siCDCA5 suppressed protein expression in all four primary cultures (Figure 5A) and inhibited the growth of these cells by 67-139%, compared to cells treated with siNT (Figure 5B).

### Clinical significance of CDCA5 expression in OSCC

**Figure 6:**
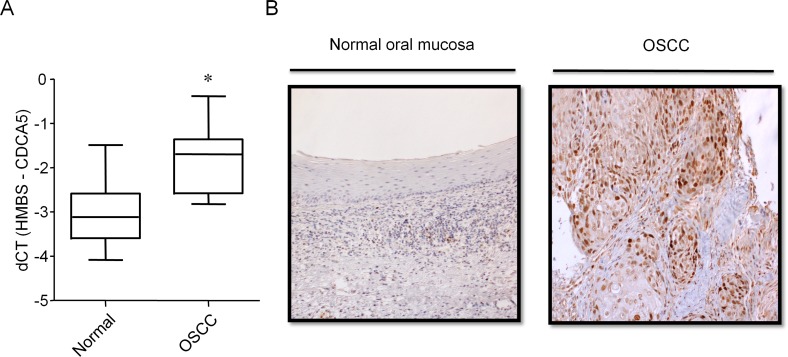
Expression of CDCA5 in OSCC tissue **A.** The expression of CDCA5 mRNA in OSCC tumors and the adjacent normal tissue from the same patient (n = 20) was analyzed by qRT-PCR using the comparative CT method and HMBS as the internal control. *, *p* < 0.01 compared to normal tissues. **B.** Sections of normal oral mucosal tissue (left panel) and OSCC tissue (right panel) labelled immunohistochemically for CDCA5 protein.

To clarify the clinical significance of CDCA5 expression, we examined the expression of CDCA5 in OSCC tissue (n = 20). The expression of CDCA5 mRNA in tumor and adjacent normal tissue derived from the same patient was examined by qRT-PCR. Expression levels of CDCA5 mRNA in OSCC tissue were significantly higher than in normal tissue (Figure 6A). We examined CDCA5 protein expression in normal oral mucosa and OSCC tissue immunohistochemically. Most of the CDCA5 expression in OSCC tissues was observed in tumor cells but not stromal cells. In normal tissues, CDCA5 expression was also detected in few lymphocytes (Figure 6B).

**Figure 7:**
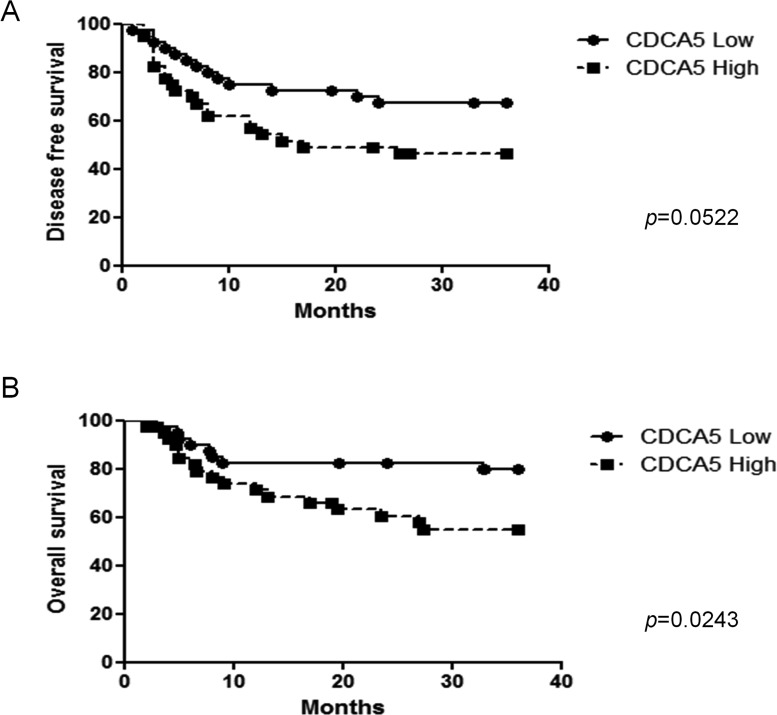
Association between CDCA5 expression level and prognosis Kaplan-Meier analysis of disease-free survival **A.** and overall survival **B.** over 3 years for patients with OSCC tumors expressing high or low CDCA5 levels.

We also evaluated the association between CDCA5 expression, assessed immunohistochemically, in tumors from 80 OSCC patients and their clinicopathological parameters. We categorized CDCA5 expression as high or low by the median value of positive rate of tumor cells, and then examined the association between CDCA5 expression and the clinicopathological parameters of the OSCC patients. Although no significant relationship was observed, high CDCA5 expression tended to be correlated with local recurrences (Table 1). Furthermore, when we examined the association between CDCA5 expression and survival by Kaplan-Meier analysis, we found that high CDCA5 expression was associated with a poor prognosis (Figure 7A, 7B).

**Table 1:** Association between CDCA5 expression in tumors from OSCC patients and their clinicopathological parameters

Parameter	Low CDCA5 (n=40) Number (%)	High CDCA5 (n=40) Number (%)	*p* value
Sex			
Male	23 (57.5)	23 (57.5)	1
Female	17 (42.5)	17 (42.5)	
Primary tumor site			
Tongue	17 (42.5)	14 (35)	0.25
Maxillary gingiva	7 (17.5)	5 (12.5)	
Mandibular gingiva	9 (22.5)	14 (35)	
Floor of mouth	1 (2.5)	4 (10)	
Buccal mucosa	5 (12.5)	3 (7.5)	
Lower lip	1 (2.5)	0 (0)	
Differentiation			
Well	33 (82.5)	24 (60)	0.05
Moderate	6 (15)	10 (25)	
Poor	1 (2.5)	6 (15)	
TNM stage			
Stage I, II	24 (60)	18 (45)	0.262
Stage III, IV	16 (40)	22 (55)	
Recurrence/Metastasis			
No	27 (67.5)	18 (42.9)	0.070
Yes	13 (32.5)	22 (57.1)	
Local recurrence	3 (7.5)	10 (25)	0.069
Lymph node metastasis	6 (15)	7 (17.5)	1
Distant metastasis	4 (10)	5 (12.5)	1

## DISCUSSION

Although CDCA5 has been reported to have roles in cell cycle progression in a variety of immortalized cell lines, through its interaction with cohesin on chromatin [8, 9], there is only one report that has investigated its possible role in carcinogenesis, in lung cancer [11]. High CDCA5 expression in lung cancer showed a significant association with a poor prognosis for patients and also promoted cell proliferation. In addition, CDCA5 was shown to be phosphorylated by extracellular signal-regulated kinase (ERK) kinase at two phosphorylation sites, Ser79 and Ser209, where consensus ERK phosphorylation site sequences, which are highly conserved in many species, were present. Ser209 phosphorylation by ERK kinase seemed to be critically important for cancer cell growth [11]. Furthermore, CDCA5 expression in peripheral T cells as well as tumor cells was associated with poor survival in lung cancer patients [10].

ERK is a member of the mitogen-activated protein kinase (MAPK) family that regulates critical phases of cell growth, including proliferation, differentiation, transcription regulation, and development [14-16]. It is well known that MAPKs play pivotal roles in carcinogenesis [17] and that the MAPK pathway is a key downstream signaling pathway regulated by epidermal growth factor receptor (EGFR) signaling in a number of cancers [14, 15]. Up to 90% of head and neck squamous cell carcinomas (HNSCC), including OSCC, are known to overexpress EGFR and this leads to excessive activation of the EGFR signaling pathway [18, 19]. Activation of EGFR-Raf-MAPK/ERK kinase-ERK signaling has been reported in several cancers, including HNSCC, and has led to the discovery of novel anticancer drugs. Recently, we have been able to use the anti-EGFR monoclonal antibody, cetuximab, to treat HNSCC patients. Compared with platinum-fluorouracil (PF) chemotherapy alone, cetuximab plus PF chemotherapy improved overall survival and progression-free survival in patients with recurrent or metastatic HNSCC, especially in OSCC patients [20]. Therefore, targeting molecules associated with ERK signaling, such as CDCA5, seems to be an appropriate approach for the treatment of OSCC.

In this study, we have demonstrated the overexpression of CDCA5 in OSCC, as well as a clinically significant correlation between CDCA5 expression and patient survival. Furthermore, we have demonstrated that targeting CDCA5 using RNA interference (RNAi) inhibited the growth of human OSCC cells both *in vitro* and *in vivo*. These results raise the possibility that CDCA5 is a novel therapeutic target for OSCC. However, at present no CDCA5-targeted drugs are available.

Our study has also demonstrated the successful transfection of tumor cell xenografts with siRNA complexed with atelocollagen [6, 21]. Atelocollagen-mediated siRNA delivery has been reported to be effective in gene silencing following either local injection directly into tumors or intravenous systemic injection. This is because siRNA complexed with atelocollagen is resistant to nuclease, so that siRNA can efficiently reach a target site *in vivo*, without being degraded by nuclease, when combined with an appropriate concentration of atelocollagen [22, 23]. In addition, our recent studies have shown that atelocollagen-mediated, systemic administration of siRNAs specific for the androgen receptor and three Akt isoforms resulted in significant growth inhibition of human prostate cancer in nude mice, without severe side effects such as lung, liver, or renal damage [24, 25]. These results suggest that nucleic acid-based drugs, such as atelocollagen-complexed siRNA, may provide novel therapeutic opportunities for human malignancies, with minimal risks of adverse events.

In summary, CDCA5 is likely to play a significant role in OSCC progression, so that targeting CDCA5 may be a potentially useful therapeutic approach for patients with OSCC.

## MATERIALS AND METHODS

### Cells and cell culture

We used four human OSCC cell lines, GFP-SAS [26], Ca9-22, HSC2, and HSC3, and an immortalized human non-neoplastic keratinocyte cell line, HaCaT, as previously described [27, 28]. All cell lines were maintained in Dulbecco's modified Eagle's medium (DMEM; Wako, Osaka, Japan) supplemented with 10% fetal bovine serum (FBS; Biosource Camarillo, CA, USA), 100 U/ml penicillin, and 100 μg/ml streptomycin (Wako), referred to here as complete medium.

Primary cell cultures were established from OSCC tumors harvested from patients. Tumor tissue was surgically excised and rinsed several times with complete medium. Each tissue sample was cut into small fragments and dissociated by treatment with 0.1% collagenase (Wako) at 37°C for 2 h. The cell suspension was filtered through a 70 μm nylon mesh cell strainer (BD, Franklin Lakes, NJ, USA). The cells were collected by centrifugation, resuspended in keratinocyte serum-free medium (K-SFM; Life Technologies, Carlsbad, CA, USA), and seeded onto plastic dishes. In K-SFM, tumor cells could continue to grow but not stromal and immune cells. All cells were grown in an incubator with a humidified atmosphere of 95% air and 5% CO_2_ at 37°C.

### Samples from patients

Twenty OSCCs with the adjacent normal tissue and 80 paraffin-embedded OSCC samples were obtained at the Ehime University Hospital between December 2001 and July 2012. Four primary cell cultures were derived from OSCCs from the lower gingiva and a lymph node metastasis (from a 55 year-old male, T4N2bM0), the lower gingiva (from a 80 year-old female, T4N1M0), and a skin metastasis (from a 61 year-old male, rT0N0M1). The Institutional Review Board (IRB) at Ehime University Hospital approved this study.

### qRT-PCR

Total RNA was extracted by lysing the cells or tissue using ISOGEN (NipponGene, Tokyo, Japan). Tissue samples were homogenized in 1.0 ml of ISOGEN using a TissueLyser (Qiagen, Valencia, CA, USA). The relative quantification of mRNA levels used the comparative threshold cycle (CT) method (ΔΔCT method), carried out by qRT-PCR using the SYBR® system. Hydroxymethylbilane synthase (HMBS) was used as internal controls. PCR amplification was carried out in 10 μl final reaction mixtures containing 5 μl 2x One Step SYBR® RT-PCR Buffer 4, 0.4 μl PrimeScript® One Step Enzyme Mix 2 (Takara, Otsu, Japan), 0.4 μl forward primer (10 μM), 0.4 μl reverse primer (10 μM), 0.2 μl ROX reference Dye II (x 50), 2.6 μl RNase-free dH_2_O, and 1 μl total RNA (100 ng/μl). The sequences of the primers used were as follows: human CDCA5, forward 5′-ATC CAC CTC GCA GGA GCC CTA-3′ and reverse 5′-CTC TCC TTC CTT GGA GCT GGA CT-3′; human HMBS, forward 5′-CAT GCA GGC TAC CAT CCA TGT-3′ and reverse 5′-GTT AGC AGT GAT GCC TAC CAA-3′. For analyzing tissue samples from mice, we used the TaqMan® system. RT-PCR was performed in a 10 μl final reaction mixture containing 5 μl 2 × Quantitect RT-PCR Master Mix, 0.1 μl Quantitect RT mix (Qiagen), 0.5 μl TaqMan® probe and primers (Life Technologies), and 100 ng total RNA. The TaqMan® probe and primers for mouse ISGF-3γ, OAS2, MX1 and HMBS were purchased from Life Technologies. The thermal-cycling conditions were reverse transcription at 42°C for 5 min and 95°C for 10 s, followed by 40 cycles at 95°C for 5 s and 60°C for 30 s. SYBR® Green I or 5′-fluorescent reporter dye fluorescence was detected with ViiA^TM7^ (Life Technologies).

### Western blot analysis

Cells (5 × 10^5^ for GFP-SAS, Ca9-22, HSC2, and HSC3) transfected with siRNAs were grown in monolayers for 48 h and then lysed in 0.5 M EDTA (Dojindo, Kumamoto, Japan) and 1% NP-40 (Nacalai Tesque, Kyoto Japan) in phosphate-buffered saline (PBS; Wako) containing a protease inhibitor cocktail and a phosphatase inhibitor (Roche Diagnostics, Basel, Switzerland). The samples were centrifuged at 15,000 g for 15 min at 4°C and the supernatants were electrophoresed on SDS–polyacrylamide gels and then transferred to polyvinylidene difluoride membranes (Millipore, Bedford, MA, USA). The membranes were blocked with 5% non-fat dried milk (Wako) in 1 × TBS-T (25 mM Tris–HCl, 125 mM NaCl, and 0.1% Tween 20 (Sigma–Aldrich, St. Louis, MO, USA)) for 1 h at room temperature. They were then probed with polyclonal rabbit anti-CDCA5 antibody (Atlas Antibodies, Stockholm, Sweden; diluted at 1:500), monoclonal mouse anti-β-tubulin antibody (BD; diluted at 1:1000), or polyclonal goat anti-actin antibody (Santa Cruz Biotechnology, Dallas, TX, USA; diluted at 1:1000) in 5% non-fat dried milk in 1 × TBS-T for 1 h at room temperature, followed by treatment with horseradish peroxidase-conjugated secondary antibodies against rabbit, mouse (GE Healthcare, Buckinghamshire, UK), or goat (SouthernBiotech, Birmingham, AL, USA) IgG for 1 h at room temperature. The immune complexes were visualized using enhanced chemiluminescence (ECL) Prime Western Blotting Detection Reagent (GE Healthcare). The density of visualized immune complexes was digitized using a RAS3000 imaging system (Fujifilm, Tokyo, Japan).

### Transfection with synthetic siRNAs

We used two siCDCA5. Synthetic siCDCA5-1 was purchased from COSMO BIO Co., Ltd. (Tokyo, Japan) with the sequence: sense 5′-CGC AGG AGC CCU AGG AUU UTT-3′ and antisense 5′-AAA UCC UAG GGC UCC UGC GTT-3′. The second sequence, siCDCA5-2, was designed by siDirect (http://sidirect2.rnai.jp): sense 5′-UCA AAC UCG GCA UUC AUG GTT-3′ and antisense 5′-UGA UCC AAG AAG UAA GUU CTT-3′. Another synthetic siNT was used as a negative control: sense 5′-UAC GUA CUA UCG CGC GGA UTT-3′ and antisense 5′-AUC CGC GCG ATA GUA CGU ATT-3′. Transfections used Lipofectamine RNAiMAX (Life Technologies) mixed with 10 nM siRNAs for Western blotting and cell proliferation assays.

### Cell growth assay

Cells were seeded into 96-well plates, using 2 × 10^3^/well for GFP-SAS and 3 × 10^3^/well for HSC2, HSC3, and Ca9-22, in complete medium with 10 nM synthetic siRNAs and 0.2% Lipofectamine RNAiMAX in a final volume of 100 μl. After 72 h, cell growth was evaluated using WST-8 assays (Cell counting Kit-8; Dojindo).

### Invasion assay

The cell invasion was measured using a Corning® BioCoat^TM^ FluoroBlok^TM^24-Multiwell Insert System (Corning, Corning, NY, USA). GFP-SAS cells were treated with siRNAs for 24 h, after which they were trypsinized, counted, and normalized for cell number between treatments. Cells (1 × 10^5^) in serum-free DMEM were placed in an insert made of polycarbonate membrane with 8-μm pores and precoated with basement membrane matrix. The outer chamber was filled with 0.5 ml of DMEM containing 5% FBS. The plate was incubated at 37°C for 24 hours. The fluorescence of the invaded cells was read at 485/535 nm with ARVO^TM^ MX 1420 Multilabel Counter (PerkinElmer, Waltham, MA, USA).

### Flow cytometry

Human OSCC cells were transfected with 10 nM synthetic siCDCA5 with Lipofectamine RNAiMAX for 48 h. After incubation, cells were detached with trypsin at 37°C, washed twice with cold PBS, and resuspended in 10 ml ice cold 70% ethanol for 2 h. The prepared cells were washed twice with PBS and then incubated at 37°C for 15 min in 0.25 mg/ml bovine pancreas ribonuclease A (Sigma-Aldrich), using 1 ml per 1 × 10^6^ cells. The suspensions were stained with propidium iodide solution (Sigma-Aldrich) at 4°C for 30 min and analyzed using an EPICS XL-MCl flow cytometer (Beckman Coulter, Fullerton, CA, USA).

### Xenograft model and tumor therapy

GFP-SAS cells were complexed with Matrigel® (BD) using 2 × 10^6^ cells per 100 μl aliquot and injected subcutaneously at two sites in the flanks of male athymic nude mice (CLEA Japan, Tokyo, Japan). One week later, tumor-bearing nude mice were randomly divided into two treatment groups, receiving either siCDCA5-1 or siGFP with the sequence: sense 5′-CUA CAA CAG CCA CAA CGU CTT-3′ and antisense 5′-GAC GUU GUG GCU GUU GUA GTT-3′. The siRNAs were used at a final concentration of 20 μM in atelocollagen (AteloGene; Koken, Tokyo, Japan). These complexes were injected into the subcutaneous spaces around the tumors every 3 days. Tumor diameters were measured at regular intervals using digital calipers and tumor volumes (mm^3^) were calculated using the formula: length × width × height × 0.523. Three mice were used in each group. Thirteen days after the first administration of siRNAs, the GFP-SAS xenografts were dissected and the CDCA5 protein expression levels were determined by western blotting. The animal studies were approved by the Ehime University animal care committee.

### Immunohistochemistry

The surgically resected OSCC specimens were fixed in 10% phosphate-buffered formalin and embedded in paraffin. A series of 4 μm thick tissue sections were prepared from each sample. Immunohistochemistry was performed using the avidin-biotin-peroxidase complex method. Briefly, the deparaffinized sections were incubated with 0.3% H_2_O_2_ in distilled water for 5 min to block endogenous peroxidase activity, and treated at 121°C in an autoclave for 20 min in 10 mM citrate buffer (pH 6.0) to regenerate epitopes. The sections were then incubated for 30 min at room temperature with anti-CDCA5 antibody (diluted 1:50; Atlas Antibodies). After washing, the sections were overlaid with biotinylated anti-rabbit antibody (Vector Laboratories, Burlingame, CA, USA) at room temperature for 30 min, washed in TBS-T, and then labeled with streptavidin-peroxidase complex (Vector Laboratories). The sections were counterstained with hematoxylin, dehydrated with ethanol, treated with xylene, and mounted in synthetic resin. We selected three hot spots for measuring labeled cells using a BIOREVO BZ-9000 microscope (Keyence, Osaka, Japan) and the prevalence of CDCA5 positive cells were calculated using Dynamic cell count BZ-H1C software (Keyence) [29]. Immunohistochemical staining for CDCA5 protein was categorized as high or low by the median value of positive rate of tumor cells.

### Statistical analysis

Student's t-test was used to determine the significance of differences between groups. Differences in patient survival were determined using the log-rank test, with *p* values < 0.05 considered statistically significant. Statistical analyses were performed using GraphPad Prism software, version 5.04 (GraphPad Software, San Diego, CA, USA).
